# C-751-05. Physical and Mental Health Recovery After Large Burn Injuries

**DOI:** 10.1093/jbcr/irag033.010

**Published:** 2026-04-06

**Authors:** Joseph Grobowski, Aditi Morumganti, Simrat Kaur, Caitlin M Orton, Xinyao deGrauw, Qian Qiu, Julie Hodapp, Lewis E Kazis, Maxwell B Johnson, Karen J Kowalske, Shelley A Wiechman, Gretchen J Carrougher, Tam N Pham, Barclay T Stewart

**Affiliations:** University of Washington Medicine Regional Burn Center, Harborview Medical Center, Seattle, WA; Tulane University, New Orleans, LA; University of Washington Medicine Regional Burn Center, Harborview Medical Center, Seattle, WA; Department of Surgery, University of Washington Medicine Regional Burn Center, Harborview Medical Center, Seattle, WA; University of Washington, Seattle, WA; University of Washington, Seattle, WA; University of Washington, Seattle, WA; Boston University School of Public Health, Boston, MA; Division of Plastic and Reconstructive Surgery, Keck School of Medicine, University of Southern California, Los Angeles, California; Department of Physical Medicine and Rehabilitation, University of Texas Southwest Medical Center, Dallas, TX; University of Washington Harborview Medical Center, Seattle, WA; University of Washington Medicine Regional Burn Center, Harborview Medical Center, Seattle, WA; University of Washington Harborview Medical Center, Seattle, WA; Department of Surgery, University of Washington School of Medicine / Harborview Injury Prevention and Research Center, Seattle, WA

## Abstract

**Introduction:**

Advances in emergency, critical and surgical care have markedly improved survival after major burn injury (≥50% TBSA), yet survivors experience major physical and psychological sequelae. We aimed to characterize long-term physical and mental health outcomes of people with burn injuries ≥50% TBSA to inform patients, families, providers and payors about expected trajectories, rehabilitation needs, functional abilities and common impairments, when compared to patients with moderate sized burn injuries (20-49.9% TBSA). We hypothesized that larger burn sizes (≥50% TBSA) would be associated with greater loss of physical function but similar mental health outcomes compared to those with more moderate burn sizes across all timepoints.

**Methods:**

Adults with burns ≥20% TBSA were identified from a multicenter longitudinal cohort study and stratified into 20–49.9%, 50–69.9% and ≥ 70% TBSA groups. Data were collected at discharge (pre-injury recall) and 6, 12, 24 and 60 months post-injury. The Short-Form-12 (SF-12), Veterans RAND-12 (VR-12) or PROMIS Global Health-10 were administered at all timepoints and linked with validated bridges to create Global Physical Health (GPH) and Global Mental Health (GMH) scores. Besides GPH and GMH, the outcomes also included SF-12 and VR-12 subdomains to explore more granular trends. Demographic and injury characteristics were summarized and mixed-effects linear regression models were used to evaluate changes in GPH, GMH and subdomain scores over time.

**Results:**

A total of 1113 participants were analyzed. GPH declined more with larger burns, with significant net differences relative to the 20–49.9% reference group (*p*<.0001; Fig. 1). GMH did not differ by burn size (Fig. 2). Subdomain analyses showed more declines with larger burns compared to the 20-49.9% group in physical function, bodily pain and role limitations due to physical problems (*p*<.05). Conversely, the ≥70% TBSA group demonstrated significant recovery in mental health at 24 months (*p*<.05).

**Conclusions:**

This analysis confirms lasting deficits in physical function, role limitations and greater bodily pain for patients with ≥50% TBSA burns. In contrast, mental health outcomes were less influenced by burn size, and people living with ≥70% TBSA burns demonstrated significant recovery at 24 months. These persistent deficits support the classification of burns as a chronic condition. Future studies should examine factors driving recovery, map findings to individualized rehabilitation plans, and visually provide prognostic information for patients, families and providers caring for those with major burn injuries.

**Applicability of Research to Practice:**

Physical and mental health recovery after 6 – 12 months of challenging rehabilitation and adaptation is the common trajectory for people with massive burn injuries. This information can help guide providers, patients and families in prognostication and formulating recommendations.

**Funding for the study:**

The contents of this abstract were developed under a grant from the National Institute on Disability, Independent Living, and Rehabilitation Research (NIDILRR grant #90DPBU0005). NIDILRR is a Center within the Administration for Community Living (ACL), Department of Health and Human Services (HHS). The contents of this abstract do not necessarily represent the policy of NIDILRR, ACL, HHS, and do not assume endorsement by the Federal Government.

